# Integrative single-cell and multi-omic analysis resolves granulosa-cell programs during chicken follicle selection

**DOI:** 10.1016/j.psj.2026.107771

**Published:** 2026-09-13

**Authors:** Ming Qu, Ji Cao, Xingjie He, Haiping Liang, Qing Wei, Jianzhen Huang, Yong Cui

**Affiliations:** College of Animal Science and Technology, Jiangxi Agricultural University, Nanchang, 330045, China

**Keywords:** Chicken, Egg-laying trait, Follicle selection, Granulosa cell, Single-cell RNA-seq

## Abstract

Follicle selection sustains the hierarchical ovulatory cycle in laying hens, yet granulosa-cell (GC) programs have not been integrated across transcriptomic, regulatory and trait-genetic datasets. We integrated GC single-cell RNA-seq with independent bulk transcriptomes, ATAC-seq, Hi-C and ChickenGTEx GWAS/eQTL resources. Integration of 62,997 cells resolved 18 fine states organized into eight major state groups and four broader programs spanning early/proliferative, pre-hierarchical/pre-selection, hierarchical/selected steroidogenic and post-ovulatory/remodeling states. Dataset-restricted analyses supported recovery of this shared state organization across sources. Slingshot ordered early/proliferative and pre-hierarchical states toward selected steroidogenic states, with early/pre-selection markers declining or peaking before steroidogenic markers increased. Across 36 independent bulk libraries, early/proliferative and DNA-replication programs were consistently depleted in large yellow follicles, whereas selected steroidogenic programs increased. Promoter accessibility and Hi-C compartment patterns provided stage-variable regulatory context. Rule-based multi-layer scoring prioritized *LHCGR, AMH* and *CYP11A1* as Tier 1 candidates. Tier 1 and Tier 2 gene sets showed stronger local ChickenGTEx GWAS signals than matched random windows. Individual candidate-window signals remained below genome-wide and chromosome-wide significance thresholds. These results establish a cross-resource GC state framework for chicken follicle selection and nominate a focused candidate set for population-specific functional validation.

## Introduction

Follicle selection is a central event in avian ovarian biology. In laying hens, follicles grow from the pre-hierarchical pool into a hierarchical ovulatory sequence, and this transition depends on coordinated changes in granulosa-cell proliferation, gonadotropin responsiveness, steroidogenic activation and post-ovulatory remodeling (Johnson and Woods, 2009; Onagbesan et al., 2009; Johnson, 2015; Huang et al., 2021). A clearer description of these granulosa-cell state transitions is therefore important for understanding how the chicken ovary maintains follicle hierarchy and reproductive output.

Several public resources now provide complementary views of chicken reproductive biology. Single-cell and multi-omic studies have described granulosa-cell heterogeneity across follicle stages, follicle selection and atresia (Li et al., 2022; Zhang et al., 2024; Yu et al., 2025). Bulk transcriptomes have captured follicle-stage and laying-age expression patterns (Sun et al., 2022), while chromatin-accessibility and three-dimensional genome datasets have begun to define regulatory landscapes in granulosa cells (Li et al., 2022; Li et al., 2026). In parallel, ChickenGTEx and related genome-wide association resources provide a useful trait-genetic context for evaluating candidate genes associated with reproductive traits (International Chicken Genome Sequencing Consortium, 2004; Liu et al., 2011; Wolc et al., 2012; Gao et al., 2022; Fu et al., 2023; Zhu et al., 2026). Together with earlier tissue-level and cell-resolved studies of the chicken ovary and reproductive axis (Wu et al., 2017; Zhu et al., 2019; Chen et al., 2021; Tai et al., 2022; Leng et al., 2024; Wang et al., 2024), these resources provide an opportunity to connect ovarian cell states with independent transcriptomic, regulatory and genetic evidence.

Several mechanistic studies also support the relevance of the candidate pathways examined here. Gonadotropin responsiveness, steroidogenic activation, SLIT-ROBO signaling and nuclear receptor activity have all been implicated in avian granulosa-cell differentiation and follicle selection (Urban et al., 1991; Johnson et al., 2002; Johnson and Lee, 2016; Ghanem and Johnson, 2018; Xu et al., 2018; Li et al., 2023; Nie et al., 2024). These observations motivated our emphasis on state-specific, trajectory-dynamic and regulatory-supported genes rather than on single-marker associations alone.

A remaining challenge is to determine whether GC programs inferred from existing single-cell atlases are reproducible across independent datasets and supported by complementary follicle-stage, regulatory and trait-genetic evidence. Existing chicken single-cell and multi-omic studies have primarily characterized cellular heterogeneity or regulatory landscapes within individual experiments, leaving the cross-dataset stability and broader biological relevance of these programs unresolved. Addressing this gap requires a cross-resource framework that distinguishes recurrent GC programs from study-specific patterns and evaluates their relevance to follicle selection and their potential relationship to reproductive traits.

In this study, we integrated public single-cell, bulk transcriptomic, chromatin and ChickenGTEx resources to construct a poultry-focused granulosa-cell state framework for chicken follicle selection (Fig. 1). We first generated an integrated granulosa-cell reference across follicle stages and inferred a selection branch from early/proliferative states toward selected steroidogenic states. We then projected single-cell-derived marker programs into an independent bulk follicle transcriptome dataset and evaluated marker-set robustness using leave-one-marker-out and expression-matched random gene-set analyses. Finally, we combined promoter-proximal ATAC, Hi-C-derived compartment and TAD context, local ChickenGTEx GWAS support and candidate-window eQTL evidence to place state-associated genes within regulatory and trait-genetic contexts. These findings provide a resource for poultry reproductive biology and for future studies of avian follicle selection, ovarian function and egg-laying traits.

**Fig. 1:**
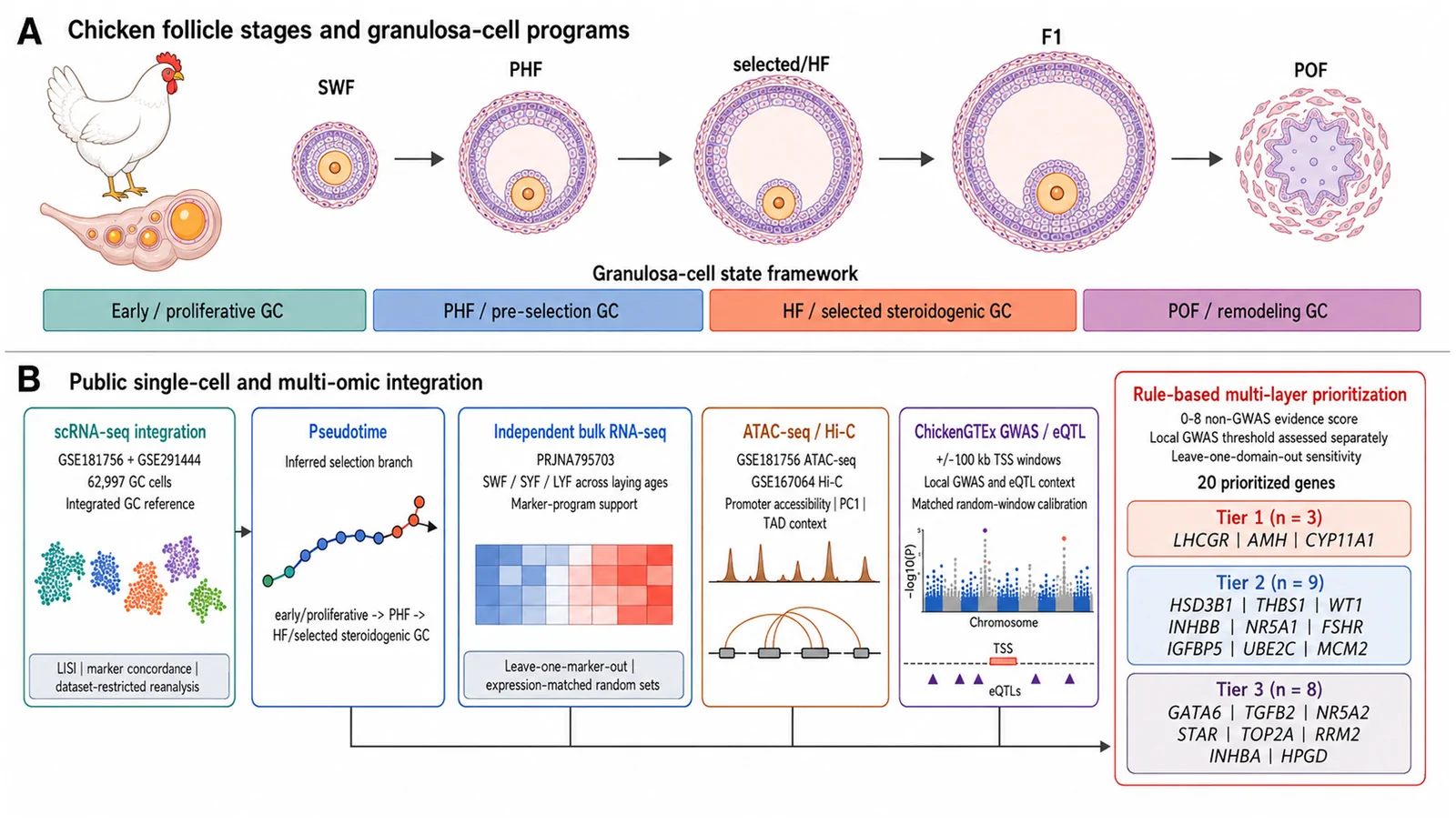
Study design for integrative granulosa-cell state analysis during chicken follicle selection. (A) Biological framework spanning small white follicles (SWF), pre-hierarchical follicles (PHF), selected/hierarchical follicles (selected/HF), the largest preovulatory follicle (F1) and post-ovulatory follicles (POF). The four analysis-level granulosa-cell (GC) programs are early/proliferative, PHF/pre-selection, HF/selected steroidogenic and POF/remodeling. The inferred selection branch terminates at HF/selected steroidogenic GC, whereas POF/remodeling represents the post-ovulatory program. (B) Cross-resource analysis and prioritization workflow. GSE181756 and GSE291444 single-cell RNA-seq data were integrated into a 62,997-cell GC reference and evaluated using local inverse Simpson's index (LISI), cross-dataset marker concordance and dataset-restricted reanalysis. The reference was used for pseudotime ordering along the inferred selection branch. State marker programs were evaluated in independent PRJNA795703 bulk follicle RNA-seq across SWF, small yellow follicles (SYF) and large yellow follicles (LYF) at three laying ages, with leave-one-marker-out and expression-matched random-set analyses. Regulatory context was assessed using GSE181756 ATAC-seq and GSE167064 Hi-C compartment PC1 and topologically associating domain (TAD) features. ChickenGTEx local GWAS windows and eQTL annotations were used as positional context, with ±100-kb windows centered on transcription start sites and matched random-window calibration. Rule-based prioritization used a 0–8 non-GWAS evidence score, assessed local GWAS support separately and tested stability by leave-one-domain-out sensitivity analysis. This framework prioritized 20 genes: Tier 1, *n* = 3 (*LHCGR, AMH* and *CYP11A1*); Tier 2, *n* = 9; and Tier 3, *n* = 8.

## Materials and methods

### Public datasets and metadata

Public sequencing resources were identified from GEO, SRA and the corresponding publications, and accession information was cross-checked before analysis (Leinonen et al., 2011; Barrett et al., 2013). Gene annotation and transcription-start-site coordinates were harmonized against Ensembl chicken gene models when analyses required genomic coordinates (Cunningham et al., 2022).

Granulosa-cell single-cell RNA-seq data were obtained from GSE181756 (Luhua hens, 31 weeks; one library each for SWF, F1 and POF) and GSE291444 (Muchuan black-bone laying hens; one deposited library each for pooled PHF and HF), respectively (Li et al., 2022; Yu et al., 2025). Independent bulk follicle RNA-seq data were obtained from PRJNA795703 (Nandan-Yao hens at 22, 31 and 51 weeks; four biological replicates per SWF, SYF and LYF age-stage group) (Sun et al., 2022). Bulk HPG-axis transcriptome data from GSE141588 were used as an exploratory high-versus-low egg-production expression context (Mishra et al., 2020). Regulatory analyses used granulosa-cell ATAC-seq peak files from GSE181756 and Hi-C-derived compartment PC1 and TAD annotations from GSE167064 (Li et al., 2022). Egg-laying-trait GWAS summary statistics and portal-derived eQTL records were obtained from the ChickenGTEx resource, which was generated in a commercial pure-line White Plymouth Rock population (Zhu et al., 2026). Dataset accessions, data types and primary analysis roles are summarized in Table S1. Expanded dataset metadata, including breed, age, tissue or follicle stage and available biological-unit or library counts, are provided in Table S2.

### Single-cell data processing and integration

The single-cell input matrices were generated from droplet-based single-cell RNA-seq resources. Quality-control and preprocessing steps followed common single-cell analysis principles implemented in the Seurat/Bioconductor ecosystem (Huber et al., 2015; Lun et al., 2016; McCarthy et al., 2017; Zheng et al., 2017). Processed 10x Genomics matrices from GSE181756 and GSE291444 were loaded and analyzed in R using Seurat (Stuart et al., 2019; Hao et al., 2024). Cells with fewer than 200 detected genes or a mitochondrial gene fraction greater than 10% were excluded.

For each sample, counts were normalized using Seurat `NormalizeData`, and 3,000 highly variable features were selected using the variance-stabilizing transformation method. Integration features were selected across samples, and all samples were merged into a single Seurat object. The merged object was scaled on the selected features and subjected to principal-component analysis with 50 principal components. Batch correction was performed with Harmony, using dataset and sample identifiers as covariates and PCA dimensions 1-40 (Korsunsky et al., 2019). UMAP embedding, nearest-neighbor graph construction and clustering were performed on the Harmony-corrected representation using dimensions 1-40. Clusters were identified at resolution 0.4.

Cluster annotation was based on marker-gene expression, cluster-specific marker tables and established GC state markers. The fine-state labels shown in Figs. 2A and 3A were mapped to seven analysis-level categories: Cycling early GC, WT1/FSHR+ PHF GC, INHBB/TGFB2+ PHF GC, Mixed/transitional GC, HF steroidogenic GC, Selected steroidogenic GC and POF remodeling GC; High-mito POF cells were classified separately as a quality-associated state. Table S3 provides the complete mapping of each fine-state label to its major category, top markers, source-dataset contribution and dominant follicle stage.

**Fig. 2:**
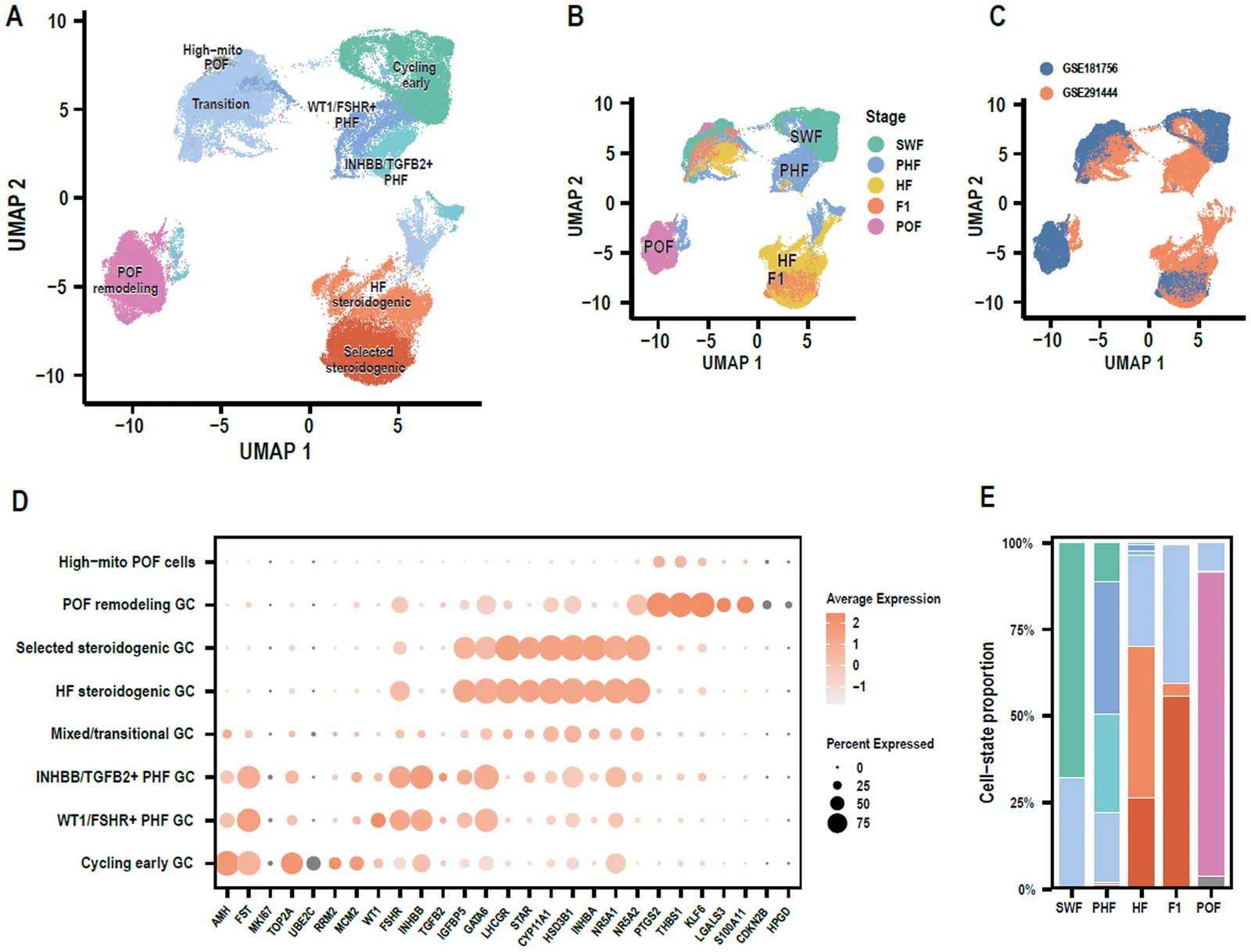
Integrated single-cell reference resolves granulosa-cell state programs across chicken follicle stages. (A) Harmony-corrected UMAP of the integrated 62,997-cell granulosa-cell reference colored by eight major state groups; labels identify the state groups. (B) The same UMAP colored by sampled follicle stage: small white follicles (SWF), pre-hierarchical follicles (PHF), hierarchical follicles (HF), F1 follicles and post-ovulatory follicles (POF). (C) The same UMAP colored by source dataset, GSE181756 or GSE291444. (D) Dot plot of marker expression across major state groups. Dot size represents the percentage of cells with detectable expression, and color represents average expression scaled within each gene across state groups. (E) Descriptive cell-state composition within each sampled stage. Each stacked bar sums to 100% of the retained cells pooled within that stage, and colors correspond to the state groups in panel A.

**Fig. 3:**
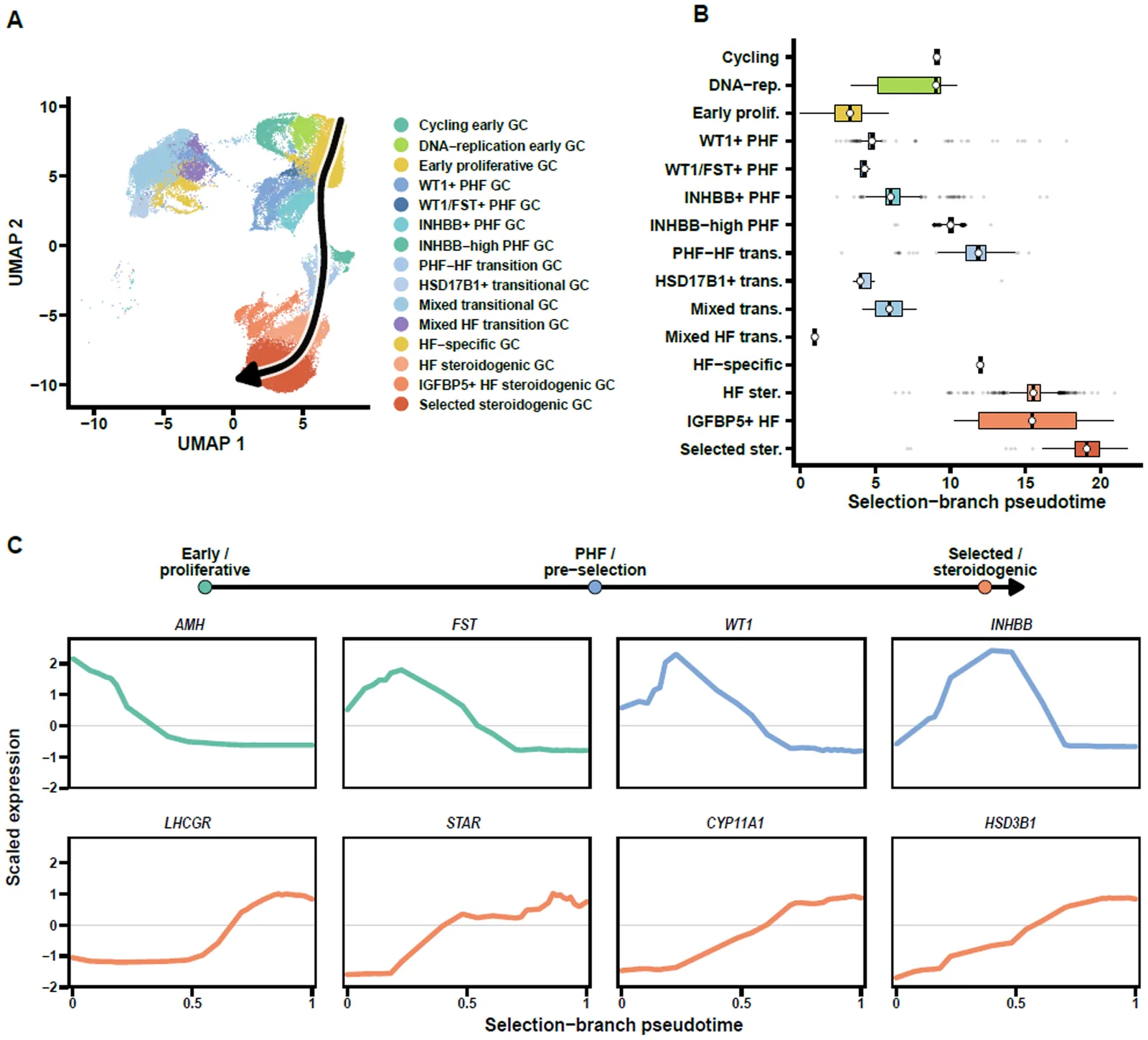
Pseudotime analysis orders granulosa-cell states along an inferred selection branch. (A) UMAP of 52,621 granulosa cells retained for trajectory analysis, colored by fine-state annotation. The black Slingshot principal curve and arrow indicate increasing pseudotime along the selected lineage, from Early proliferative GC toward Selected steroidogenic GC. (B) Selection-branch pseudotime distributions for the 24,930 cells assigned a nonmissing value on this lineage. Boxes span the interquartile range, center lines and superimposed white points indicate medians, whiskers extend to 1.5 times the interquartile range, and gray points show values beyond the whiskers. Box colors correspond to the fine-state colors in panel A. (C) Binned expression dynamics of representative markers along the selected lineage. The 24,195 cells with lineage weight >0.2 were divided into 25 pseudotime quantile bins. Points show mean normalized expression within each bin and connecting lines show the resulting temporal profiles. Pseudotime was rescaled from 0 to 1, and expression was z-scaled within each gene. Green denotes early/proliferative markers *AMH* and *FST*, blue denotes PHF/pre-selection markers *WT1* and *INHBB*, and orange denotes selected/steroidogenic markers *LHCGR, STAR, CYP11A1* and *HSD3B1*.

Integration performance was evaluated because dataset, breed, age and sampled follicle stage were partially confounded. LISI was calculated on a deterministic 30,000-cell subsample in the first 40 principal-component dimensions before Harmony and in the first 40 Harmony dimensions after correction (perplexity 30). Dataset and sample iLISI quantified local source mixing, whereas state cLISI assessed preservation of annotated cell-state separation. Within-state dataset iLISI was additionally estimated only for states containing at least 100 cells from each source dataset. Cross-dataset marker concordance was calculated as the Spearman correlation of state-versus-rest expression effects across 1,530 genes present in all five split RNA data layers, together with overlap among the top 100 positive markers. Finally, each source dataset was independently normalized, dimension-reduced, reclustered and compared with the integrated-reference labels using adjusted Rand index (ARI), normalized mutual information (NMI) and cluster purity. Unified integration and reproducibility metrics are reported in Table S4.

### Cell-state annotation and marker analysis

Cluster marker genes were identified using Seurat marker-detection functions (Stuart et al., 2019; Hao et al., 2024). Marker expression was summarized at the annotated cell-state level and visualized with dot plots, in which dot size represented the fraction of cells expressing a marker and color represented scaled average expression. Cell-state composition was calculated as the fraction of cells assigned to each annotated state within each follicle stage. Sample- and follicle-stage-specific cell counts after annotation are provided in Table S5.

### Pseudotime inference of the follicle-selection trajectory

To infer an ordering of granulosa-cell states along a putative follicle-selection branch, the annotated Seurat object was subset to states representing the early/proliferative, PHF-like, PHF-HF transitional, HF steroidogenic and selected steroidogenic continuum. POF-dominated remodeling states were not used as the endpoint of the main selection branch. The subset was converted to a SingleCellExperiment object (Amezquita et al., 2020), and Slingshot was applied using the integrated UMAP embedding and annotated cell-state labels (Street et al., 2018). The start cluster was set to Early proliferative GC when present, with Cycling early GC used as a fallback label in compatible annotated objects.

Slingshot lineage pseudotime and lineage weights were extracted for each cell. The main biologically interpretable lineage connecting early/proliferative, PHF-like, PHF-HF transitional, HF steroidogenic and selected steroidogenic states was selected for downstream visualization. Pseudotime distributions were summarized by annotated cell state. Marker-gene dynamics along pseudotime were calculated using cells with lineage weight greater than 0.2. Cells were binned into 25 pseudotime bins, normalized expression was averaged within each bin and marker values were scaled gene-wise for visualization.

### Bulk RNA-seq quantification

Raw RNA-seq runs from PRJNA795703 were downloaded with SRA Toolkit from the NCBI Sequence Read Archive (Leinonen et al., 2011). FASTQ files were quantified using Salmon against the chicken reference transcriptome (Patro et al., 2017). Transcript-level estimates were summarized to gene-level TPM and count matrices using the project quantification scripts. Samples were grouped by laying age and follicle stage according to the PRJNA795703 sample metadata.

### Bulk marker-program scoring

Single-cell-derived marker programs were projected into PRJNA795703 bulk follicle RNA-seq samples. Marker genes were mapped to the gene-level TPM matrix using gene symbols and project-level Ensembl identifier mappings where available. Expression values were transformed as log2(TPM + 1). For each detected marker gene, expression was standardized across all bulk samples. Marker-program scores were calculated as the mean standardized expression of detected marker genes in each program.

Marker-program scores were summarized by laying age and follicle stage. Pairwise comparisons were performed within each age group for follicle-stage contrasts and within each follicle stage for age contrasts. Multiple-testing correction was performed using the Benjamini-Hochberg false-discovery-rate procedure (Benjamini and Hochberg, 1995). Sample-level marker-program scores and detected marker counts are provided in Table S6.

### Marker-program robustness analysis

Two sensitivity analyses were performed to assess whether PRJNA795703 bulk marker-program trends depended on individual markers or on arbitrary marker-set composition. First, each marker program was rescored after removing one detected marker gene at a time. Follicle-stage contrasts were recalculated within each laying age, and leave-one-marker-out LYF-versus-SWF deltas were compared with the full marker-set delta. Second, expression-matched random gene sets were generated as background controls. All genes in the PRJNA795703 TPM matrix were transformed as log2(TPM + 1), standardized gene-wise across samples and binned into 20 bins by mean expression. For each observed marker gene, a random gene was sampled from the same expression bin while excluding observed marker genes. For each marker program, 1,000 expression-matched random gene sets containing the same number of detected genes were generated. Observed LYF-versus-SWF marker-program deltas were compared with the corresponding random-gene-set distributions using one-sided empirical *P* values with a one-count correction. The robustness analysis used three criteria: at least 80% of leave-one-marker-out contrasts retained the full-set direction; at least 80% retained at least 75% of the full-set absolute effect size; and the random-background empirical *P* value was ≤ 0.05. A contrast was designated stringent-robust only when all three criteria were met. Detailed leave-one-marker-out, expression-matched random-background and marker-gene-mapping outputs are provided in Table S7; criterion-specific stringent-robust calls are summarized in Table S8.

### State-module and candidate-gene definition

State-module genes were defined from the integrated single-cell annotation, pseudotime analysis and bulk projection results. Four main biological modules were used: early/proliferative, PHF/pre-selection, HF/selected steroidogenic and POF/remodeling. Candidate-gene lists used for regulatory and genetic prioritization were manually constrained to genes with interpretable granulosa-cell state, trajectory, bulk, regulatory or trait-genetic support.

### Promoter-proximal ATAC-seq analysis

Promoter-proximal chromatin accessibility was assessed using GSE181756 granulosa-cell ATAC-seq peak files. Ensembl gene annotation was used to define transcription start sites. Promoter windows were defined as ±2 kb around each annotated TSS. Peak files were parsed into genomic intervals and intersected with promoter windows using genomic range operations (Lawrence et al., 2013). For each candidate gene and state module, we recorded whether at least one promoter-overlapping ATAC peak was present in SWF, F1 or POF peak calls. Module-level promoter support was summarized as the number and fraction of module genes with promoter-overlapping peaks in each follicle stage.

### Hi-C compartment and TAD analysis

GSE167064 Hi-C-derived compartment PC1 and TAD annotation files were parsed for SWF, F1 and POF samples. Candidate-gene transcription start sites were mapped to 20 kb compartment PC1 bins. For each state module, PC1 values were summarized across genes and follicle stages. TAD support was assessed by determining whether each candidate-gene TSS fell within an annotated TAD and whether it was located within 20 kb of a TAD boundary. Gene- and module-level regulatory-support summaries, including promoter ATAC overlap, compartment PC1 and TAD context, are provided in Table S9.

### Candidate-gene scoring and tier assignment

Candidate genes were prioritized using an explicit eight-point matrix organized into six non-GWAS evidence domains. State-module membership, pseudotime dynamics and independent bulk follicle-stage support each contributed 0 or 1 point. Promoter ATAC contributed one point for promoter overlap and one point for concordance with the expected follicle stage. Compartment PC1 likewise contributed one point each for PC1 dynamics and expected-stage concordance, whereas TAD-boundary proximity contributed 0 or 1 point. The resulting non-GWAS evidence score ranged from 0 to 8. A domain was counted as supporting when its domain score was greater than 0, giving 0-6 supporting non-GWAS domains. Portal-derived eQTL overlap was tabulated separately and did not contribute a tier point.

Local ChickenGTEx GWAS evidence was treated as a seventh domain and represented by the strongest local -log10(*P*), rather than being added to the eight-point score. Tier 1 required a core mechanistic gene, score ≥6, support from ≥4 of the 6 non-GWAS domains and best local -log10(*P*) ≥3. Tier 2 required support from ≥4 non-GWAS domains and local -log10(*P*) ≥2.5, together with either a core mechanistic gene with score ≥5 or any gene with score ≥6. Tier 3 required score ≥5 and support from ≥4 non-GWAS domains without meeting Tier 1 or Tier 2 local-GWAS criteria; genes with score <5 or support from <4 domains were retained as exploratory. The full per-domain score matrix and leave-one-domain-out sensitivity are reported in Table S10. When the ATAC or PC1 domain was omitted, both component criteria were removed from the score.

### Local GWAS window analysis using ChickenGTEx data

Public GWAS summary statistics were obtained from the associated figshare repository for six ChickenGTEx traits: age at first egg (AFE), body weight at 49 days (BW49), egg number at 210 days (EN210), egg number at 300 days (EN300), egg number at 400 days (EN400), and egg number between 210 and 400 days (EN210-400) (Zhu et al., 2026). For each candidate gene, variants within a fixed ±100 kb window centered on the annotated TSS were scanned across all six traits. The minimum *P* value, maximum -log10(*P*), local lead variant and number of variants within each window were recorded. The source GWAS and eQTL resources were generated in a commercial pure-line White Plymouth Rock population. The ±100 kb interval was applied uniformly to permit cross-gene comparison and was not intended to represent a biological boundary, population-specific LD decay distance or LD block. Candidate-window signals were treated as suggestive positional evidence unless they reached a reported genome-wide or chromosome-wide significance threshold. Individual-level genotypes and a population-specific LD matrix were unavailable; therefore, population-specific LD decay and locus-specific SNP-SNP pairwise *r*^2^ could not be estimated. Because *r*^2^ is defined between pairs of genetic variants, it is not applicable to the relationship between a SNP and a fixed TSS coordinate.

### Matched random-window enrichment analysis

Matched random windows were generated for each candidate-gene window to test whether local GWAS support around prioritized genes exceeded random expectation. Chromosome bounds were inferred from the ChickenGTEx GWAS summary statistics. For each candidate gene, 300 random windows were sampled on the same chromosome with the same window size as the candidate TSS-centered window, excluding windows overlapping any candidate-gene window on the same chromosome.

For each random window, the minimum *P* value and maximum -log10(*P*) were calculated across the same six ChickenGTEx traits used for candidate-gene windows. For candidate-gene empirical tests, random windows matched to each gene were ranked by similarity in local SNP count, and the 120 closest windows were used to calculate one-sided empirical *P* values with a one-count correction. For tier-level enrichment, the best observed local GWAS signal for each gene across traits was compared with 2,000 permutations in which each gene was assigned a random-window signal sampled from its SNP-count-matched background. Tier-level empirical *P* values were calculated as the proportion of permuted mean best -log10(*P*) values greater than or equal to the observed tier mean, again using a one-count correction. Candidate-window GWAS results for all gene-trait combinations are provided in Table S11.

### ChickenGTEx eQTL query and candidate-window overlap analysis

To distinguish portal-wide from window-restricted support, eQTL coordinates were parsed from ChickenGTEx portal-derived variant identifiers and intersected with the fixed ±100 kb candidate windows. For each candidate, we recorded the strongest portal eQTL variant and coordinate, the strongest eQTL retained within the candidate window, tissue context, *P* value, distance to the local GWAS lead, whether the strongest portal eQTL lay within the window and whether the local GWAS lead itself appeared as an eQTL. Pairwise LD *r*^2^ between a local GWAS lead and an eQTL variant could not be estimated because population-matched individual genotypes or a suitable LD reference panel were unavailable; *r*^2^ between a SNP and the fixed gene TSS coordinate is undefined. Because matched full tissue-specific eQTL summary statistics and allele-harmonization inputs were also unavailable, formal statistical colocalization was not performed. These overlaps were therefore treated only as positional annotations.

### Exploratory expression analysis by egg-production group

GSE141588 HPG-axis bulk RNA-seq data were used as an exploratory expression context for prioritized candidate genes. The GEO-provided FPKM matrix was parsed, and sample names were used to assign tissue, individual and high- or low-production group metadata. Candidate genes from the final prioritization table were mapped to the matrix using ChickenGTEx-resolved Ensembl gene identifiers and project-level fallback mappings. Expression values were transformed as log2(FPKM + 1), standardized gene-wise across samples and summarized as module scores by averaging standardized expression across detected candidate genes within each module.

High- versus low-production comparisons were performed separately within ovary, pituitary gland and hypothalamus samples. Candidate-gene and module-level effect sizes were reported as high-minus-low differences in standardized expression or module score. Welch-type tests were used for exploratory *P* values, and *P* values were adjusted using the Benjamini-Hochberg method within the generated result tables.

### Locus-level visualization of prioritized candidates

For six high-priority candidates (*AMH, LHCGR, CYP11A1, HSD3B1, WT1* and *INHBB*), SNP-level summary statistics for the ChickenGTEx trait showing the strongest local GWAS signal were visualized within a ±250 kb region centered on the TSS. The formal ±100 kb candidate window, candidate-window eQTL variants and stage-specific promoter-proximal ATAC peaks from GSE181756 were overlaid. eQTL variants were displayed as positional annotations rather than evidence of statistical colocalization, and plots were generated in R using ggplot2, patchwork and related tidyverse packages (Wickham, 2016; Wickham et al., 2019).

## Results

### An integrated single-cell reference resolves granulosa-cell state programs across chicken follicle stages

Integration of GSE181756 and GSE291444 yielded a 62,997-cell granulosa-cell reference spanning five follicle stages (Fig. 2A-C). The reference comprised 35,058 cells from GSE181756 and 27,939 cells from GSE291444. It included 13,441 SWF, 12,711 PHF, 15,228 HF, 11,108 F1 and 10,509 POF cells (Table S5). Unsupervised clustering resolved 18 fine states, which were consolidated into eight major state groups for summary analyses (Fig. 2A; Table S3).

In a fixed 30,000-cell subset used for integration diagnostics, Harmony modestly increased source mixing without eroding state separation. Mean dataset iLISI rose from 1.004 to 1.145, and mean sample iLISI rose from 1.010 to 1.244. Median dataset iLISI remained at 1.000, whereas median sample iLISI increased from 1.000 to 1.033. Mean state cLISI changed only from 1.193 to 1.175 (Table S4).

Among states represented in both datasets, state-versus-rest marker effects showed moderate concordance. Spearman correlations ranged from 0.445 to 0.721 across nine fine states and from 0.326 to 0.699 across five major states, with a median of 0.597 at both levels. Dataset-restricted reclustering also recovered the reference organization. Fine-state ARI/NMI/purity values were 0.744/0.766/0.882 for GSE181756 and 0.629/0.722/0.814 for GSE291444; major-state purity was 0.949 and 0.880, respectively (Table S4). Together, these diagnostics supported recovery of the shared state organization in two datasets with distinct breed and follicle-stage compositions.

Marker expression distinguished the eight major state groups (Fig. 2D). Cycling early GC expressed cell-cycle-associated markers including *TOP2A, UBE2C, RRM2* and *MCM2. WT1*/*FSHR*+ PHF GC and *INHBB*/*TGFB2*+ PHF GC expressed *AMH, FST, WT1, FSHR, INHBB* and *TGFB2*. HF steroidogenic GC and Selected steroidogenic GC were enriched for *LHCGR, STAR, CYP11A1, HSD3B1, INHBA, NR5A1* and *NR5A2*. POF remodeling GC expressed *PTGS2, THBS1, KLF6, LGALS3* and *S100A11*.

The major state groups showed distinct stage-structured distributions (Fig. 2E; Table S5). SWF cells were dominated by Cycling early GC, whereas PHF cells were enriched for *WT1*/*FSHR*+ PHF GC and *INHBB*/*TGFB2*+ PHF GC. HF cells comprised Mixed/transitional GC, HF steroidogenic GC and Selected steroidogenic GC, whereas F1 cells were dominated by Selected steroidogenic GC. POF cells mainly comprised POF remodeling GC. Because the two datasets sampled non-overlapping follicle stages, Fig. 2E summarizes pooled cell composition within each stage rather than differential abundance across datasets.

### Pseudotime analysis orders granulosa-cell states along an inferred follicle-selection branch

Slingshot analysis retained 52,621 granulosa cells for trajectory inference. With Early proliferative GC specified as the root, the selected lineage assigned nonmissing pseudotime values to 24,930 cells and ordered PHF-like and transitional states before HF and selected steroidogenic states (Fig. 3A and B). Median pseudotime was 3.31 in Early proliferative GC, 4.77 in *WT1*+ PHF GC and 6.02 in *INHBB*+ PHF GC. It increased to 10.00 in *INHBB*-high PHF GC, 11.87 in PHF-HF transition GC, 15.52 in HF steroidogenic GC and 19.09 in Selected steroidogenic GC.

Marker dynamics were evaluated in 24,195 cells with lineage weight > 0.2. *AMH* decreased across pseudotime, whereas *FST, WT1* and *INHBB* reached their highest expression in early-to-intermediate pseudotime bins. *LHCGR, STAR, CYP11A1* and *HSD3B1* increased toward late pseudotime (Fig. 3C). Together, the state ordering and marker dynamics delineated a transcriptional transition from early/pre-selection programs toward selected steroidogenic programs.

### Independent bulk follicle transcriptomes support stage-dependent granulosa-cell marker programs

To assess whether the single-cell-defined state programs were recapitulated independently, we analyzed 36 bulk RNA-seq libraries from SWF, SYF and LYF follicles at W22, W31 and W51, with four biological replicates per age-stage group (Sun et al., 2022). Marker-program scores separated samples primarily according to follicle stage (Fig. 4A and B; Table S6).

**Fig. 4:**
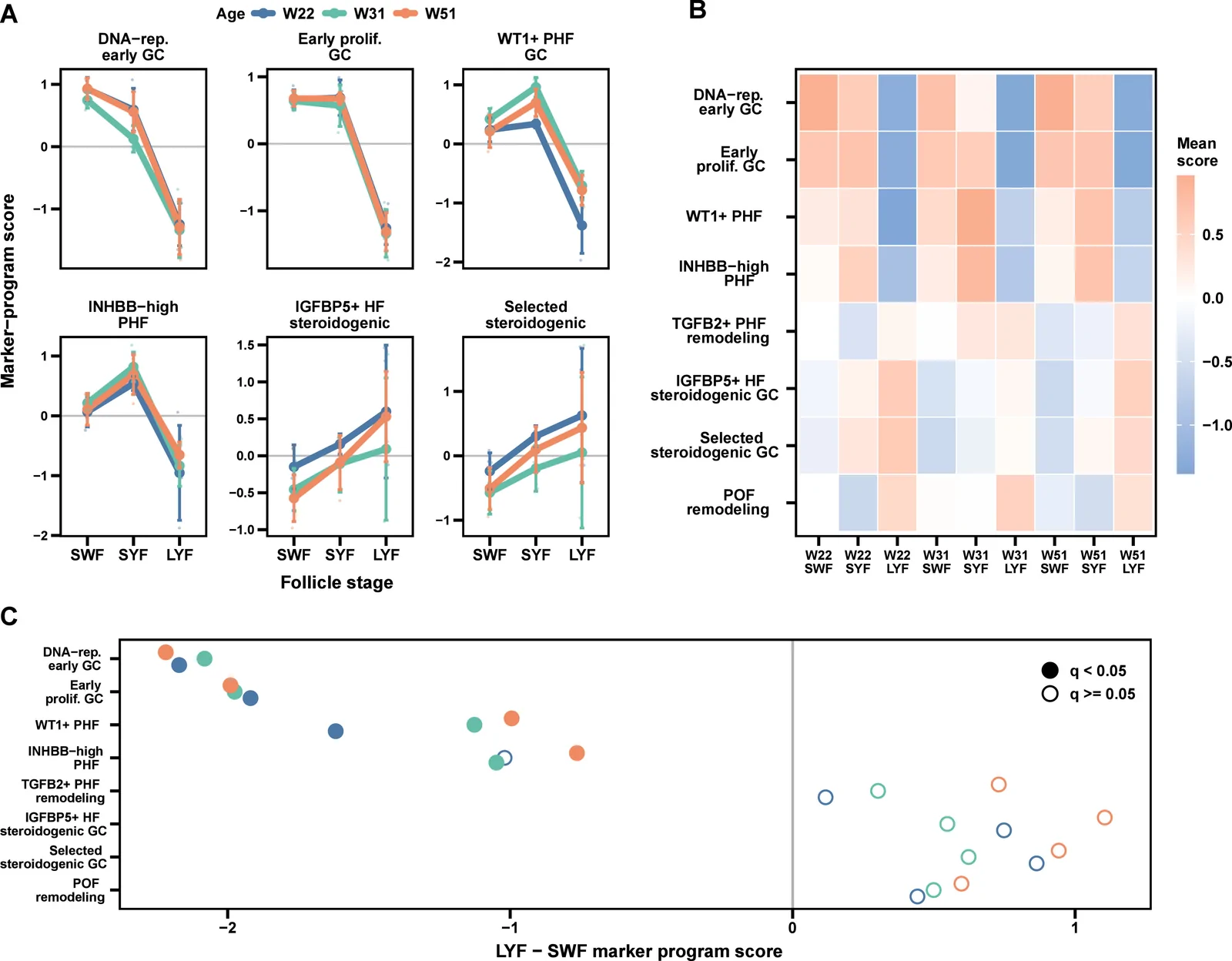
Independent bulk follicle transcriptomes support stage-dependent granulosa-cell marker programs. (A) Marker-program scores for six representative programs across 36 PRJNA795703 bulk follicle RNA-seq libraries (*n* = 4 biological replicates per age-stage group). SWF, small white follicles; SYF, small yellow follicles; LYF, large yellow follicles. W22, W31 and W51 indicate hens at 22, 31 and 51 weeks of age, respectively. Small points show individual libraries; large points and connecting lines show group means, and error bars indicate mean ± SD. Blue, green and orange denote W22, W31 and W51, respectively. (B) Heatmap of group mean scores for all eight programs across the nine age-stage groups; blue, white and orange indicate lower, near-zero and higher scores, respectively. (C) Mean LYF-minus-SWF score differences within each age. Negative values indicate lower scores in LYF, whereas positive values indicate higher scores. Colors denote age as defined in panel A; filled and open points indicate Benjamini-Hochberg-adjusted *q* < 0.05 and *q* ≥ 0.05, respectively. *P* values were obtained using two-sided Welch's *t*-tests and adjusted across all within-age stage comparisons using the Benjamini-Hochberg procedure.

The most consistent pattern was depletion of early/proliferative programs in LYF follicles. Early proliferative GC scores were significantly lower in LYF than in SWF at W22, W31 and W51 after Benjamini-Hochberg correction (adjusted *P* = 0.0016, 0.0034 and 0.0019, respectively). DNA-replication early GC scores showed the same pattern (adjusted *P* = 0.0019, 0.0066 and 0.0040, respectively). WT1-positive PHF GC signatures also decreased in LYF relative to SWF or SYF, consistent with reduced representation of a pre-hierarchical/pre-selection program in large yellow follicles. Conversely, Selected steroidogenic, IGFBP5-positive HF steroidogenic and remodeling signatures generally increased toward LYF, although these shifts were less consistent than the depletion of early/proliferative programs (Fig. 4C). No within-stage comparisons among laying ages remained significant after multiple-testing correction, indicating that follicle stage was the predominant axis captured by these programs.

Leave-one-marker-out analysis showed that the Early proliferative GC and DNA-replication early GC programs retained the full-set direction and at least 75% of the full-set absolute effect under every perturbation at W22, W31 and W51. Selected steroidogenic GC and IGFBP5-positive HF steroidogenic GC met the criteria that at least 80% of perturbations retained the full-set direction and at least 80% retained at least 75% of the full-set effect at W22 and W51, but not at W31. POF remodeling GC retained its direction but did not meet this stability criterion at any age (Fig. S1A; Tables S7 and S8).

Across the eight marker programs tested for LYF versus SWF, the proportions satisfying the direction-retention, 75% effect-retention and empirical-background criteria were 87.5%, 75.0% and 75.0% at W22; 87.5%, 50.0% and 62.5% at W31; and 100%, 62.5% and 75.0% at W51, respectively (Fig. S1B and C). The corresponding proportions satisfying all three criteria were 50.0%, 25.0% and 50.0%. Only Early proliferative GC and DNA-replication early GC met all three criteria at every age, whereas Selected steroidogenic GC and IGFBP5-positive HF steroidogenic GC met them at W22 and W51. Criterion-specific results for every age, program and contrast are provided in Table S8.

### Chromatin accessibility and 3D-genome features provide regulatory context for state-associated modules

Promoter accessibility varied among granulosa-cell state modules and follicle stages (Fig. 5A-C; Table S9). Early/proliferative genes showed promoter-overlapping ATAC peaks for 4 of 7 genes in SWF and F1, compared with 2 of 7 genes in POF. Promoter accessibility was less frequent for the PHF/pre-selection module, involving 1 of 6 genes in SWF and POF and 2 of 6 genes in F1. The HF/selected steroidogenic module showed its greatest promoter coverage in F1, with peaks detected for 4 of 7 genes in SWF, 6 of 7 genes in F1 and 5 of 7 genes in POF. By contrast, the POF/remodeling module showed its greatest coverage in POF, where 6 of 7 genes had promoter-overlapping peaks, compared with 4 of 7 genes in SWF and F1.

**Fig. 5:**
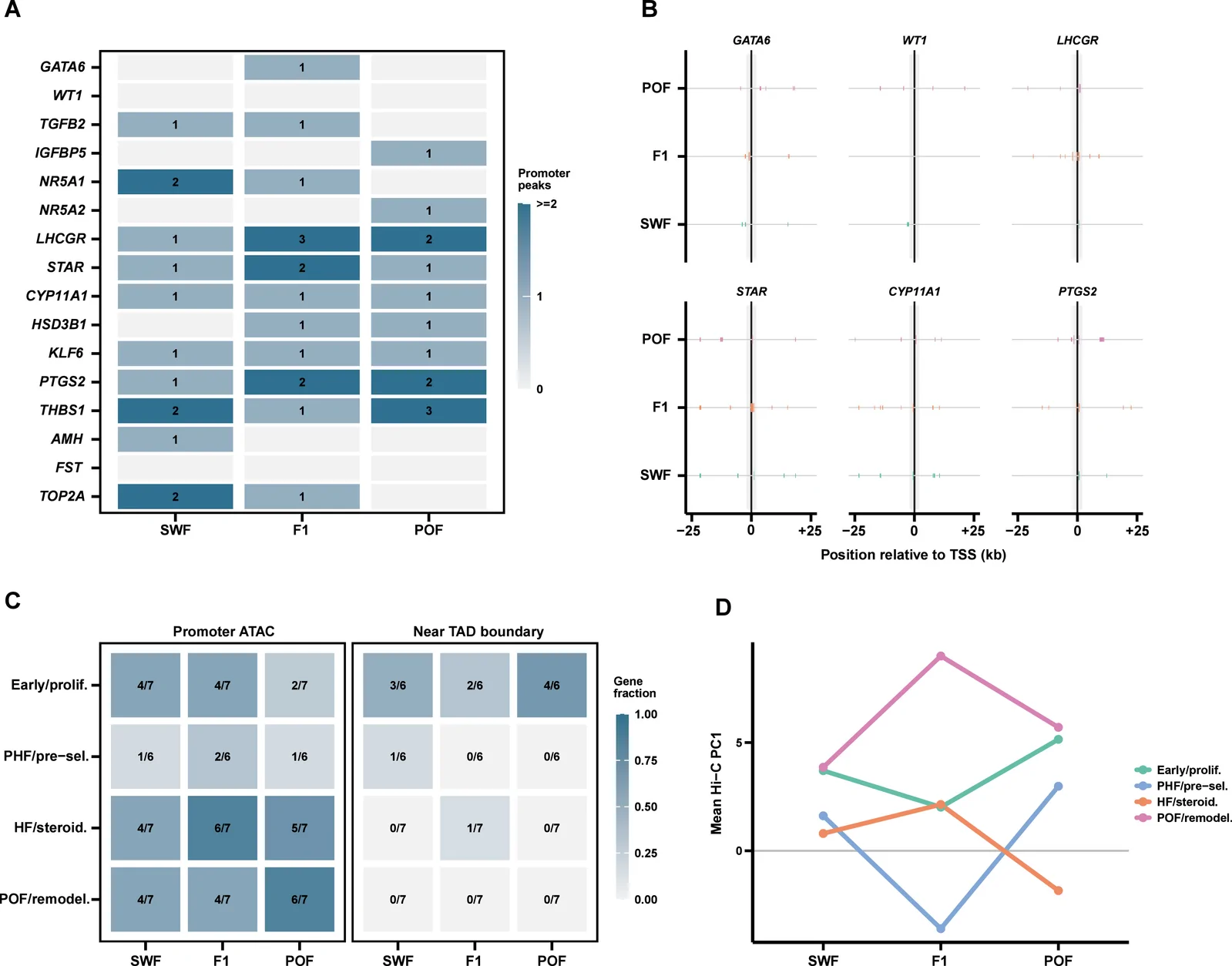
Chromatin accessibility and 3D-genome features provide regulatory context for state-associated modules. (A) Promoter-proximal ATAC support at 16 candidate genes in granulosa-cell peak calls from small white follicles (SWF), F1 follicles and post-ovulatory follicles (POF). Promoters were defined as ±2 kb around the annotated transcription start site (TSS). Numbers within tiles show the exact number of promoter-overlapping peaks; gray indicates no overlap, and the darkest fill represents ≥2 peaks. (B) ATAC peaks within ±25 kb of the TSS at six selected loci. Black vertical lines mark the TSS, gray shading marks the ±2 kb promoter window, and green, orange and magenta segments denote SWF, F1 and POF peaks, respectively. Thicker segments overlap the promoter window. (C) Module-level fractions of genes with at least one promoter-overlapping ATAC peak (left) or with a TSS located within 20 kb of a topologically associating domain (TAD) boundary (right). Tile labels give the number of supported genes divided by the number of genes assessed, and fill intensity represents the corresponding fraction. (D) Mean Hi-C compartment PC1 values for 20-kb bins containing the TSSs of genes in the early/proliferative, PHF/pre-selection, HF/steroidogenic and POF/remodeling modules. Points show the module mean at each follicle stage, lines connect stage means within a module, colors identify modules, and the horizontal gray line marks PC1 = 0.

Hi-C-derived compartment PC1 and TAD annotations provided complementary genomic context (Fig. 5C and D; Table S9). Mean PC1 for the HF/selected steroidogenic module was highest in F1 (2.14), compared with SWF (0.80) and POF (-1.84), whereas the POF/remodeling module showed higher mean PC1 values in F1 (9.00) and POF (5.70) than in SWF (3.86). Most module genes were located within annotated TADs across stages. Proximity to TAD boundaries was concentrated in the early/proliferative module, involving 3 of 6 genes in SWF, 2 of 6 genes in F1 and 4 of 6 genes in POF. Together, these patterns place the state-associated modules within stage-variable promoter-accessibility and 3D-genome contexts.

### Multi-omic prioritization places granulosa-cell state-associated genes in reproductive trait-genetic context

An explicit scoring framework integrated state-module membership, pseudotime dynamics, independent bulk validation, promoter accessibility, compartment PC1 and TAD-boundary proximity into an eight-point non-GWAS score (Fig. 6A; Table S10). Local ChickenGTEx GWAS evidence was evaluated separately for tier assignment (Fig. 6B and C). *LHCGR* scored 7 of 8 across five non-GWAS domains, whereas *AMH* and *CYP11A1* each scored 6 of 8 across five domains. These three core genes met the Tier 1 criteria; nine other genes were assigned to Tier 2 and eight to Tier 3.

**Fig. 6:**
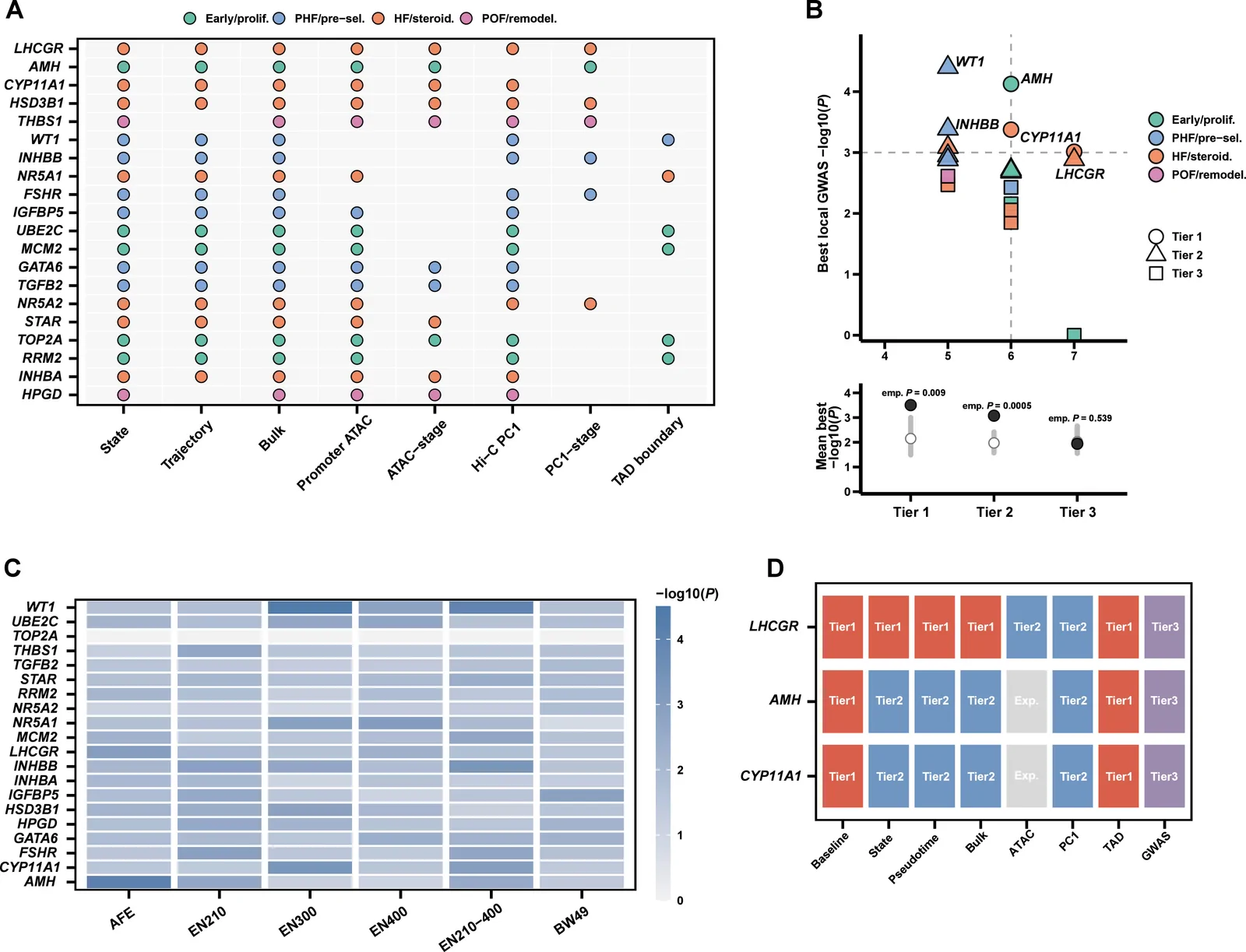
Multi-omic candidate prioritization places granulosa-cell state-associated genes in reproductive trait-genetic context. (A) Binary non-GWAS evidence matrix for the 20 prioritized genes, ordered by baseline tier and colored by granulosa-cell module. Filled circles indicate that a criterion was met. Columns represent State, state-module membership; Trajectory, pseudotime dynamics; Bulk, independent bulk follicle-stage support; Promoter ATAC, promoter-overlapping ATAC peaks; ATAC-stage, concordance between promoter accessibility and the expected follicle stage; Hi-C PC1, compartment PC1 dynamics; PC1-stage, concordance between PC1 and the expected stage; and TAD boundary, a transcription start site within 20 kb of an annotated TAD boundary. (B) Rule-based candidate prioritization using the 0-8 non-GWAS evidence score and the strongest candidate-window ChickenGTEx signal within a fixed ±100 kb TSS-centered window. In the upper panel, the x-axis shows the non-GWAS evidence score and the y-axis shows the strongest local -log10(*P*); colors denote granulosa-cell modules and shapes denote candidate tiers. Dashed guides mark the Tier 1 numerical cutoffs; complete tier rules additionally require core-gene status and support from at least four non-GWAS domains. The lower panel compares observed tier means with SNP-count-matched random-window distributions; empirical *P* values were calculated from 2,000 permutations. (C) Heatmap of the maximum candidate-window -log10(*P*) for each gene and trait. Columns represent AFE, age at first egg; EN210, EN300 and EN400, egg number at 210, 300 and 400 days, respectively; EN210-400, egg number between 210 and 400 days; and BW49, body weight at 49 days. (D) Leave-one-domain-out sensitivity of the three baseline Tier 1 genes. Baseline denotes the tier obtained from the complete evidence set; State, Pseudotime, Bulk, ATAC, PC1, TAD and GWAS denote tiers recalculated after individually omitting the indicated evidence domain. ATAC and PC1 omissions removed both subcriteria within the corresponding domain. Exp. denotes an exploratory assignment.

Leave-one-domain-out analysis distinguished the stability of the three Tier 1 assignments (Fig. 6D; Table S10). *LHCGR* remained Tier 1 after removal of state-module, pseudotime, bulk or TAD-boundary evidence and shifted to Tier 2 after removal of ATAC or PC1 evidence. *AMH* and *CYP11A1* retained Tier 1 only after omission of TAD-boundary evidence, which contributed no baseline point to either gene. Removing any supported non-GWAS domain lowered both assignments, and removing local GWAS evidence placed all three genes in Tier 3. Across these perturbations, *LHCGR* had the most stable Tier 1 evidence profile.

Matched random-window analysis tested whether tier-level local GWAS signals exceeded those expected for windows with comparable SNP counts. Tier 1 and Tier 2 candidates had stronger mean local signals than matched windows (empirical *P* = 0.0090 and 0.00050, respectively; Fig. 6B), whereas Tier 3 candidates showed no enrichment (empirical *P* = 0.539). None of the individual candidate-window lead signals reached the reported genome-wide or chromosome-wide significance thresholds. The enrichment observed specifically in Tier 1 and Tier 2 provided candidate-set-level support for the local GWAS component of the ranking.

Candidate-window scans identified distinct reproductive-trait signals for the three Tier 1 genes (Fig. 6C; Table S12). *AMH* had the strongest Tier 1 signal, with 28_2209821_G_A for AFE (-log10(*P*) = 4.126), 98,112 bp from its TSS. For *CYP11A1*, the local lead was 10_3033943_T_A for EN300 (-log10(*P*) = 3.376), 19,653 bp from its TSS. For *LHCGR*, the local lead was 3_8220811_A_G for AFE (-log10(*P*) = 3.013), 10,209 bp from its TSS.

ChickenGTEx eQTL records extended the locus profiles across tissues (Figs. S2 and S3; Table S12). *AMH* and *CYP11A1* had in-window eQTL variants 28_2201561_T_C and 10_3056142_G_A, located 8,260 and 22,199 bp from their respective GWAS leads. The strongest portal eQTL for *LHCGR*, 3_9226085_G_A in heart, was 1,005,274 bp from the GWAS lead, and no in-window eQTL was identified. None of the 19 available local GWAS leads was itself recorded as an eQTL. Population-matched LD data were unavailable, precluding pairwise *r*^2^ estimation and formal colocalization. Together, these records defined distinct trait-genetic profiles for the three Tier 1 genes within the common scoring framework.

An additional analysis of GSE141588 compared module and candidate-gene expression between high- and low-egg-production chickens (Fig. S4). No candidate module or gene reached Benjamini-Hochberg-adjusted significance. *HPGD* showed nominally higher standardized expression in high-production ovaries (high-minus-low difference = 0.167, *P* = 0.014), but the adjusted *P* value was 0.682. No other candidate gene or module had a nominal *P* < 0.05. With 2-3 samples per group, this dataset was treated as exploratory and was not used for tier assignment. Across the integrated analyses, *LHCGR, AMH* and *CYP11A1* remained the leading candidates, supported by complementary cell-state, regulatory and reproductive trait-genetic evidence.

## Discussion

Previous chicken studies described GC heterogeneity and the transcriptional and chromatin remodeling accompanying folliculogenesis (Li et al., 2022; Yu et al., 2025). Stage-resolved bulk profiles and ChickenGTEx subsequently provided complementary expression and trait-genetic resources (Sun et al., 2022; Zhu et al., 2026). By integrating these datasets, our study delineates a coherent continuum of chicken GC states across follicle development and shows that the corresponding transcriptional programs recur across complementary expression resources. Incorporating chromatin and trait-genetic evidence further identifies stage-associated regulatory modules and prioritizes candidate genes in the context of follicle selection and egg-laying traits.

The resolved GC states reveal a marked remodeling of granulosa-cell identity during follicle selection. Proliferative and prehierarchical programs predominated before selection, whereas selected follicles acquired differentiated steroidogenic programs, consistent with established models of avian follicle development (Johnson and Woods, 2009; Johnson, 2015). The same transition was recovered in both single-cell datasets and supported by independent bulk follicle profiles, reinforcing its biological consistency across complementary transcriptomic resources. Age-related differences were concentrated in the strength of later steroidogenic programs, indicating modulation of steroidogenic activation within an otherwise stable differentiation pattern.

The chromatin data place these transcriptional state transitions within a broader program of regulatory remodeling. Promoter accessibility was more prevalent among genes associated with HF and selected steroidogenic states in F1 and among remodeling genes in POF. These stage-matched patterns indicate that activation of the respective programs coincides with changes in local chromatin accessibility. Stage-associated shifts in compartment PC1 and TAD context extended this correspondence to higher-order genome organization. The convergence of promoter accessibility, compartment organization and TAD context with expression therefore supports coordinated chromatin remodeling during GC differentiation.

Among the prioritized genes, *LHCGR, CYP11A1* and *AMH* capture complementary aspects of GC differentiation and reproductive-trait biology. *LHCGR* variants have been associated with age at first egg and egg number in Erlang Mountain chickens (Li et al., 2013), while *CYP11A1* was identified as an egg-number candidate in Shuanglian chickens (Fu et al., 2023). *AMH* showed a different evidence profile: its Val566Ile variant was not associated with egg number, and its expression did not differ between Lueyang black-boned and Nick chickens in the same study (Dang et al., 2020). In our analysis, however, *AMH* marked a prehierarchical GC program and received additional regulatory support. Thus, *LHCGR* and *CYP11A1* draw support from reported trait associations, whereas *AMH* is prioritized by its state-specific expression and regulatory context during follicle selection.

Matched random-window analysis showed that Tier 1 and Tier 2 candidates carried stronger local GWAS signals than comparable genomic regions, indicating a non-random concentration of trait-associated variation around the prioritized genes. No individual signal reached the reported genome-wide or chromosome-wide thresholds, so the genetic evidence is most informative as locus-level context. *LHCGR* remains highly prioritized by its strong cellular and chromatin evidence, although the absence of an in-window eQTL leaves its local regulatory route unresolved. The distinct GWAS lead and eQTL variants at *AMH* and *CYP11A1* similarly place their current support at the locus level and motivate variant-resolved follow-up. Together, these loci define focused targets for testing how GC-state regulation contributes to reproductive variation.

Use of public resources enabled analysis across developmental stages and molecular layers that would be difficult to assemble in a single experiment. Because the source studies differed in breed, age and follicle sampling, some source effects remain intertwined with biological differences. The agreement among dataset-restricted analyses, state-marker concordance, pseudotime ordering and independent bulk profiles nevertheless supports the major proliferative-to-steroidogenic transition. For the genetic component, the standardized ±100 kb screen and matched random-window analysis provide a reproducible basis for locus prioritization within the White Plymouth Rock population represented in ChickenGTEx. Population-matched LD and colocalization data will refine the relevant variants and intervals.

The resulting candidate set offers a practical route from GC-state biology to population-level evaluation. Targeted genotyping in breed-matched cohorts can test associations with age at first egg, egg number and laying persistency. Integrating validated variants with local LD, haplotypes and existing genomic-selection models can determine whether they add predictive value for breeding. In parallel, cell-based functional studies can clarify how prioritized genes shape GC differentiation and follicle selection. This progression from state-resolved discovery to population validation provides a focused strategy for developing biologically informed reproductive markers in chickens.

## Ethics approval

No new animal experiments were performed in this study. All analyses were based on publicly available datasets.

## Data availability

No new sequencing data were generated in this study. The publicly available datasets reanalyzed here are available from the NCBI Gene Expression Omnibus under accessions GSE181756, GSE291444, GSE141588 and GSE167064, and from the NCBI Sequence Read Archive under BioProject accession PRJNA795703. ChickenGTEx GWAS summary statistics for AFE, BW49, EN210, EN300, EN400 and EN210-400 are available from figshare at https://doi.org/10.6084/m9.figshare.28333362.v1. Candidate-gene eQTL records were retrieved from the ChickenGTEx portal (https://ngdc.cncb.ac.cn/chickengtex/) .

## Financial support

This work was supported by the National Natural Science Foundation of China (No. 32402851, 32360867, 31960690, and 32503004).

## Author contributions

**Ming Qu** led single-cell data integration, cell-state annotation and pseudotime analysis; integrated the main results; prepared the core figures; and drafted the corresponding Methods and Results sections.

**Ji Cao** led analytical workflow development, quality control and robustness testing; independently checked key results; and contributed substantive revisions to the Methods and Results sections. Ji Cao also provided funding support for this study.

**Xingjie He** was responsible for public-data acquisition and bulk RNA-seq analysis, performed the associated statistical comparisons, and contributed to result interpretation and manuscript revision.

**Haiping Liang** contributed to study design and bulk RNA-seq analysis, with an emphasis on comparisons across follicle stages and their biological interpretation, and revised the manuscript.

**Qing Wei** contributed to study design and data acquisition; screened datasets, verified sample metadata and documented data sources; and revised the manuscript.

**Jianzhen Huang** contributed to the study concept and methodological design, provided scientific guidance, critically assessed the biological interpretation of key results, and substantively revised the manuscript. Jianzhen Huang also provided funding support for this study.

**Yong Cui** coordinated the study concept and overall analytical strategy, contributed to key analyses and cross-omic interpretation, and led preparation of the first draft, manuscript revision and finalization. Yong Cui also provided funding support for this study.

All authors critically reviewed the manuscript for important intellectual content, have reviewed and explicitly approved the exact final version for publication, and agree to be personally and publicly accountable for all aspects of the work, including ensuring that any questions concerning its accuracy or integrity are thoroughly investigated and resolved.

Fig. S1. Marker-score robustness for bulk follicle validation.

(A) Leave-one-marker-out analysis of LYF-versus-SWF marker-program score differences across W22, W31 and W51 follicles. Open circles show the full marker-set delta and horizontal intervals show the range after dropping one marker at a time. (B) Observed deltas compared with expression-matched random gene-set backgrounds. (C) One-sided empirical support from 1,000 random sets. Criterion-specific results under the requirement that at least 80% of perturbations retain at least 75% of the full-set effect are reported in Table S8.

Fig. S2. ChickenGTEx eQTL annotations for prioritized candidate genes.

Rows are ordered by prioritization tier and granulosa-cell state module. Colored cells show the strongest ChickenGTEx eQTL -log10(*P*) value for each gene-tissue pair; blank cells indicate that no portal eQTL record was retained for that pair.

Fig. S3. Locus-level visualization of top candidate genes.

SNP-level local ChickenGTEx GWAS signals, eQTL variants and promoter ATAC context around *AMH, LHCGR, CYP11A1, HSD3B1, WT1* and *INHBB*. Each panel shows the strongest local trait for the corresponding gene across a ±250 kb visualization window centered on the candidate-gene transcription start site. The grey shaded region marks the formal ±100 kb candidate window used for Fig. 6 prioritization, and the dashed line marks the TSS. Open circles indicate the local GWAS lead variant. Downward black triangles indicate general candidate-window eQTL variants, whereas upward black triangles indicate candidate-window eQTL variants from HPG-axis or liver contexts. Colored tracks at the bottom show SWF, F1 and POF promoter-region ATAC peaks from GSE181756. Only called ATAC peaks within the displayed TSS-centered window are drawn; absent colored tracks indicate no peak call in that stage-window combination.

Fig. S4. Exploratory high-versus-low egg-production expression analysis in GSE141588.

(A) Candidate module scores in GSE141588 HPG-axis bulk RNA-seq samples, grouped by ovary, pituitary gland and hypothalamus and by high- or low-egg-production status. Scores were calculated from detected candidate genes in each module after gene-wise standardization of log2(FPKM + 1) expression. (B) High-minus-low effect sizes for detected candidate modules across HPG-axis tissues. Values indicate mean score differences between high- and low-production groups. (C) High-minus-low effect sizes for the 16 priority genes detected in GSE141588. Filled dots indicate nominal *P* < 0.05, and asterisks indicate Benjamini-Hochberg-adjusted *P* < 0.05. *HPGD* in ovary was the only gene-level comparison reaching nominal significance (*P* = 0.014; adjusted *P* = 0.682); no gene-level comparison remained significant after adjustment.

Table S1. Public datasets, accessions, data types and primary analysis roles used in this study.

Table S2. Metadata for public datasets used in this study.

Table S3. Mapping between fine granulosa-cell states and major states used for summary analyses.

Table S4. Unified single-cell integration and reproducibility metrics, including global LISI summaries, within-state dataset iLISI, cross-dataset marker-effect concordance and dataset-restricted reclustering.

Table S5. Cluster- and stage-level cell-count summaries.

Table S6. Bulk follicle marker-program scores.

Table S7. Detailed marker-score robustness outputs, including leave-one-marker-out results, expression-matched random-background tests and marker-gene mapping.

Table S8. Criterion-specific marker-score robustness results using an 80% perturbation-frequency requirement and a 75% effect-retention threshold.

Table S9. Unified regulatory-support metrics for stage-associated granulosa-cell state modules, including promoter ATAC overlap, compartment PC1 and TAD context.

Table S10. Candidate-tier definitions, baseline domain scores, local GWAS context and leave-one-domain-out sensitivity, including recalculated scores and tiers after omission of each evidence domain.

Table S11. Candidate-window ChickenGTEx GWAS results for the 20 prioritized genes across six traits.

Table S12. Candidate local GWAS and ChickenGTEx eQTL annotations for the 20 prioritized genes.

## Disclosures

All authors declare that they have no competing interests.

## Footnotes

Supplementary material associated with this article can be found, in the online version, at doi:10.1016/j.psj.2026.107771.
