# Supplementary material for: Integrative single-cell and multi-omic analysis resolves granulosa-cell programs during chicken follicle selection
Source: Poult Sci. 2026 Sep 13;105(12):107771. doi: 10.1016/j.psj.2026.107771 (PMC13631913; doi:10.1016/j.psj.2026.107771)
Supplement: Supplementary file 1 [file mmc1.pdf]

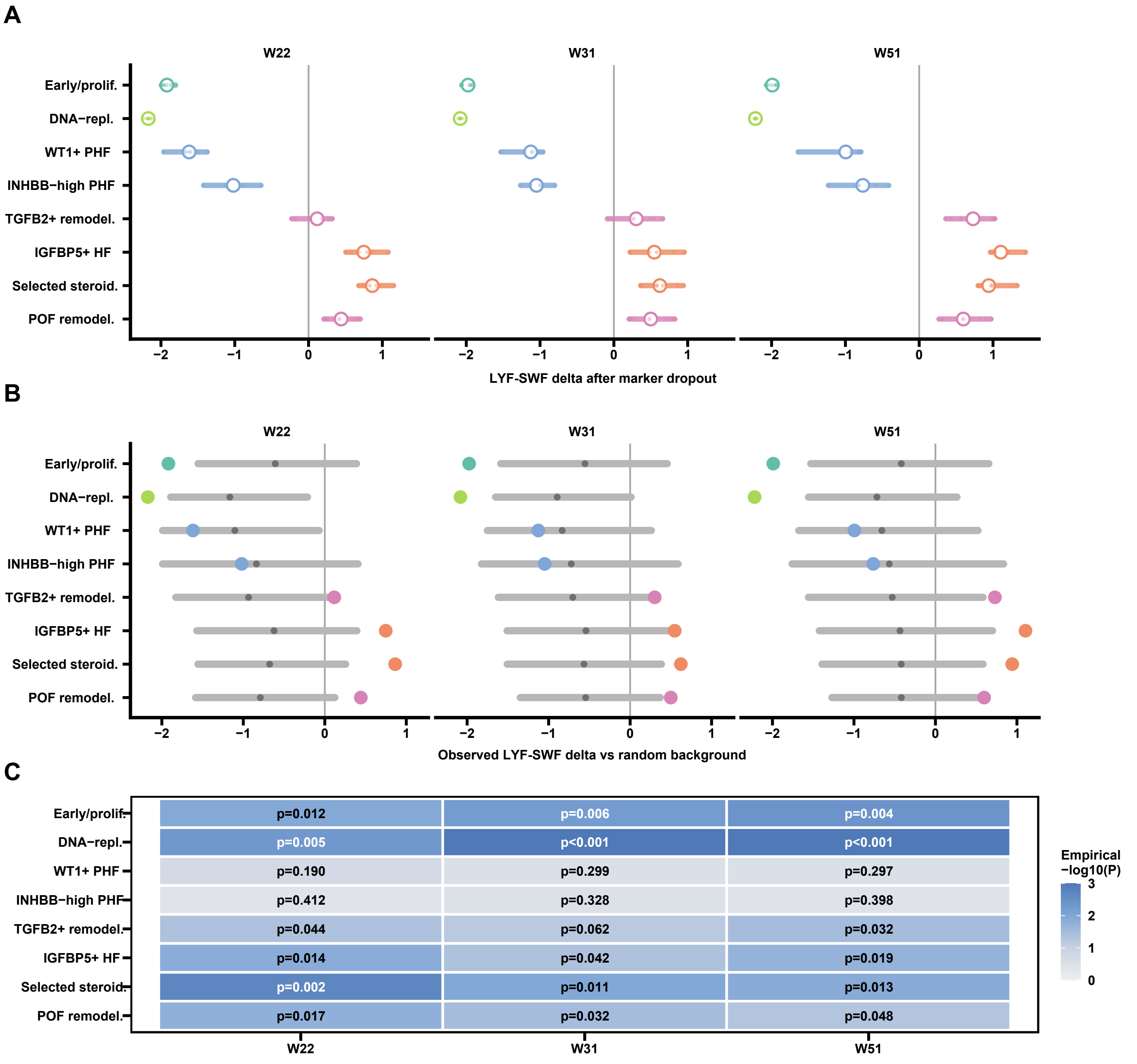
A
W22
W31
W51
Early/prolif.
DNA−repl.
WT1+ PHF
INHBB−high PHF
TGFB2+ remodel.
IGFBP5+ HF
Selected steroid.
POF remodel.
−2
−1
0
1
LYF-SWF delta after marker dropout
B
W22
W31
W51
Early/prolif.
DNA−repl.
WT1+ PHF
INHBB−high PHF
TGFB2+ remodel.
IGFBP5+ HF
Selected steroid.
POF remodel.
−2
−1
0
1
Observed LYF-SWF delta vs random background
C
Early/prolif. p=0.012 p=0.006 p=0.004
DNA−repl. p=0.005 p<0.001 p<0.001
WT1+ PHF p=0.190 p=0.299 p=0.297
INHBB−high PHF p=0.412 p=0.328 p=0.398
TGFB2+ remodel. p=0.044 p=0.062 p=0.032
IGFBP5+ HF p=0.014 p=0.042 p=0.019
Selected steroid. p=0.002 p=0.011 p=0.013
POF remodel. p=0.017 p=0.032 p=0.048
W22
W31
W51
Empirical
−log10(P)
3
2
1
0
