# Supplementary material for: Integrative single-cell and multi-omic analysis resolves granulosa-cell programs during chicken follicle selection
Source: Poult Sci. 2026 Sep 13;105(12):107771. doi: 10.1016/j.psj.2026.107771 (PMC13631913; doi:10.1016/j.psj.2026.107771)
Supplement: Supplementary file 2 [file mmc2.pdf]

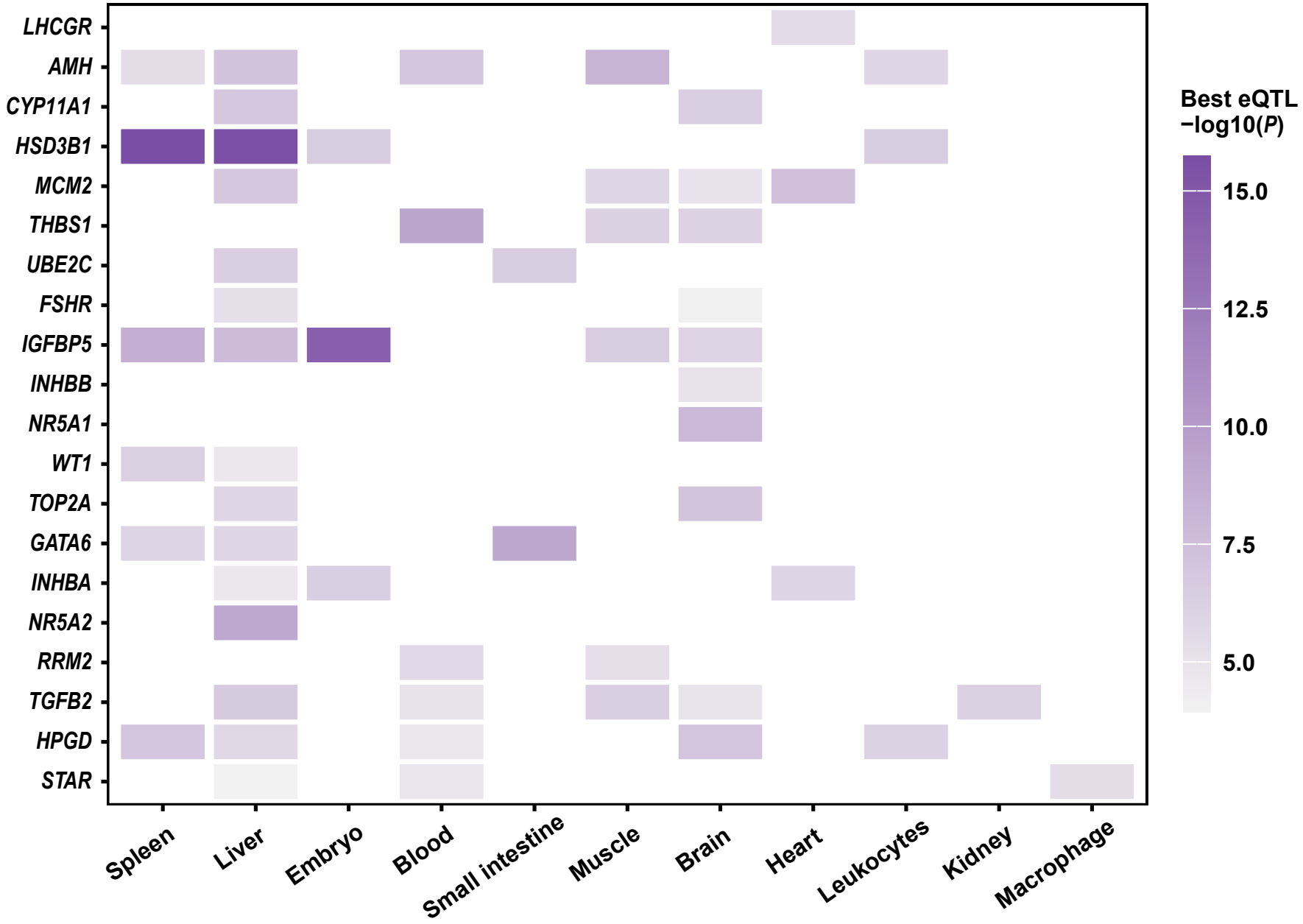

LHCGR
AMH
CYP11A1
HSD3B1
MCM2
THBS1
UBE2C
FSHR
IGFBP5
INHBB
NR5A1
WT1
TOP2A
GATA6
INHBA
NR5A2
RRM2
TGFB2
HPGD
STAR
Spleen
Liver
Embryo
Blood
Small intestine
Muscle
Brain
Heart
Leukocytes
Kidney
Macrophage
Best eQTL
−log10(P)
15.0
12.5
10.0
7.5
5.0
