# Supplementary material for: Integrative single-cell and multi-omic analysis resolves granulosa-cell programs during chicken follicle selection
Source: Poult Sci. 2026 Sep 13;105(12):107771. doi: 10.1016/j.psj.2026.107771 (PMC13631913; doi:10.1016/j.psj.2026.107771)
Supplement: Supplementary file 3 [file mmc3.pdf]

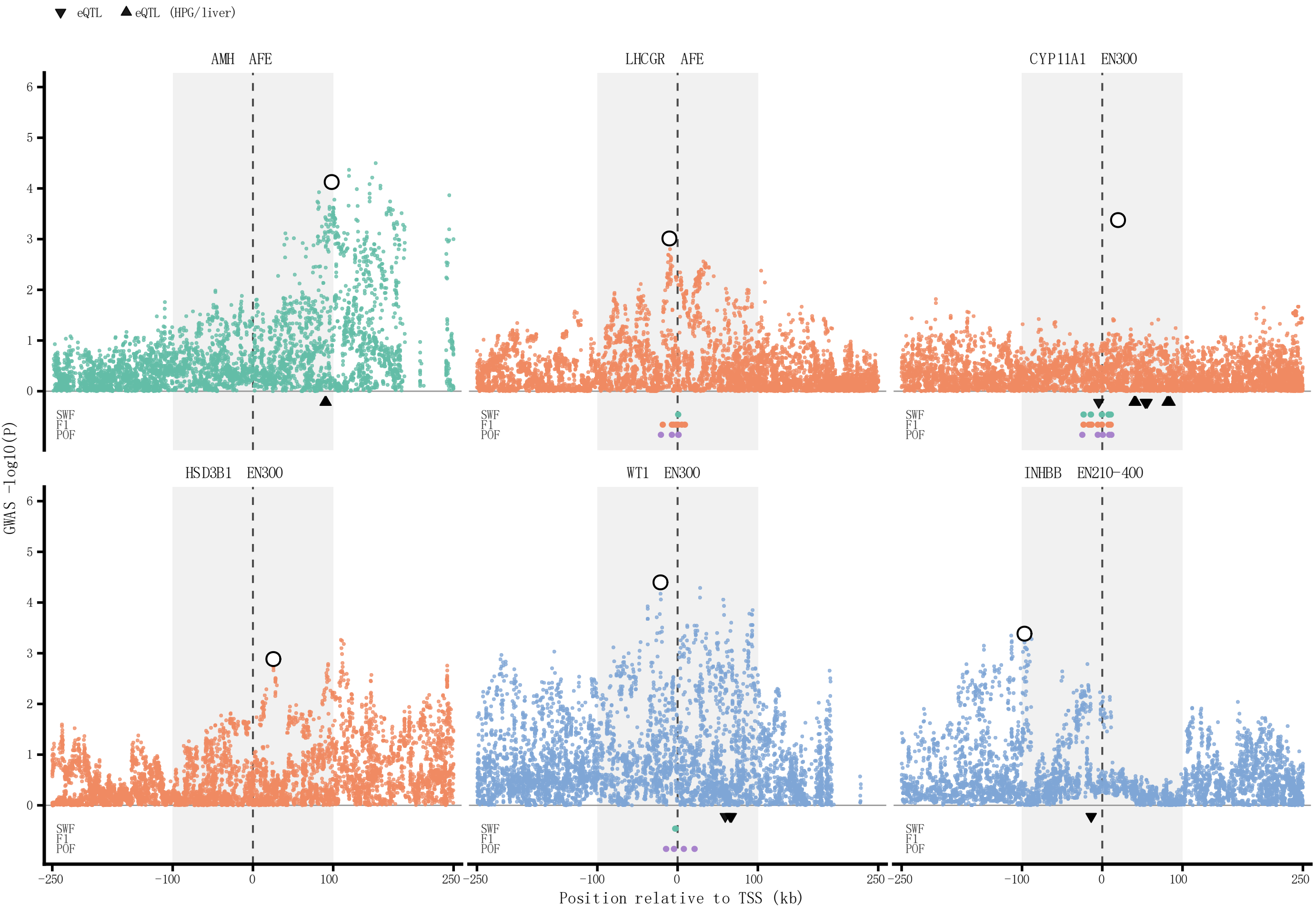

eQTL
eQTL (HPG/liver)
AMH AFE
LHCGR AFE
CYP11A1 EN300
HSD3B1 EN300
WT1 EN300
INHBB EN210-400
SWF
F1
POF
GWAS -log10(P)
Position relative to TSS (kb)
