# Supplementary material for: Integrative single-cell and multi-omic analysis resolves granulosa-cell programs during chicken follicle selection
Source: Poult Sci. 2026 Sep 13;105(12):107771. doi: 10.1016/j.psj.2026.107771 (PMC13631913; doi:10.1016/j.psj.2026.107771)
Supplement: Supplementary file 4 [file mmc4.pdf]

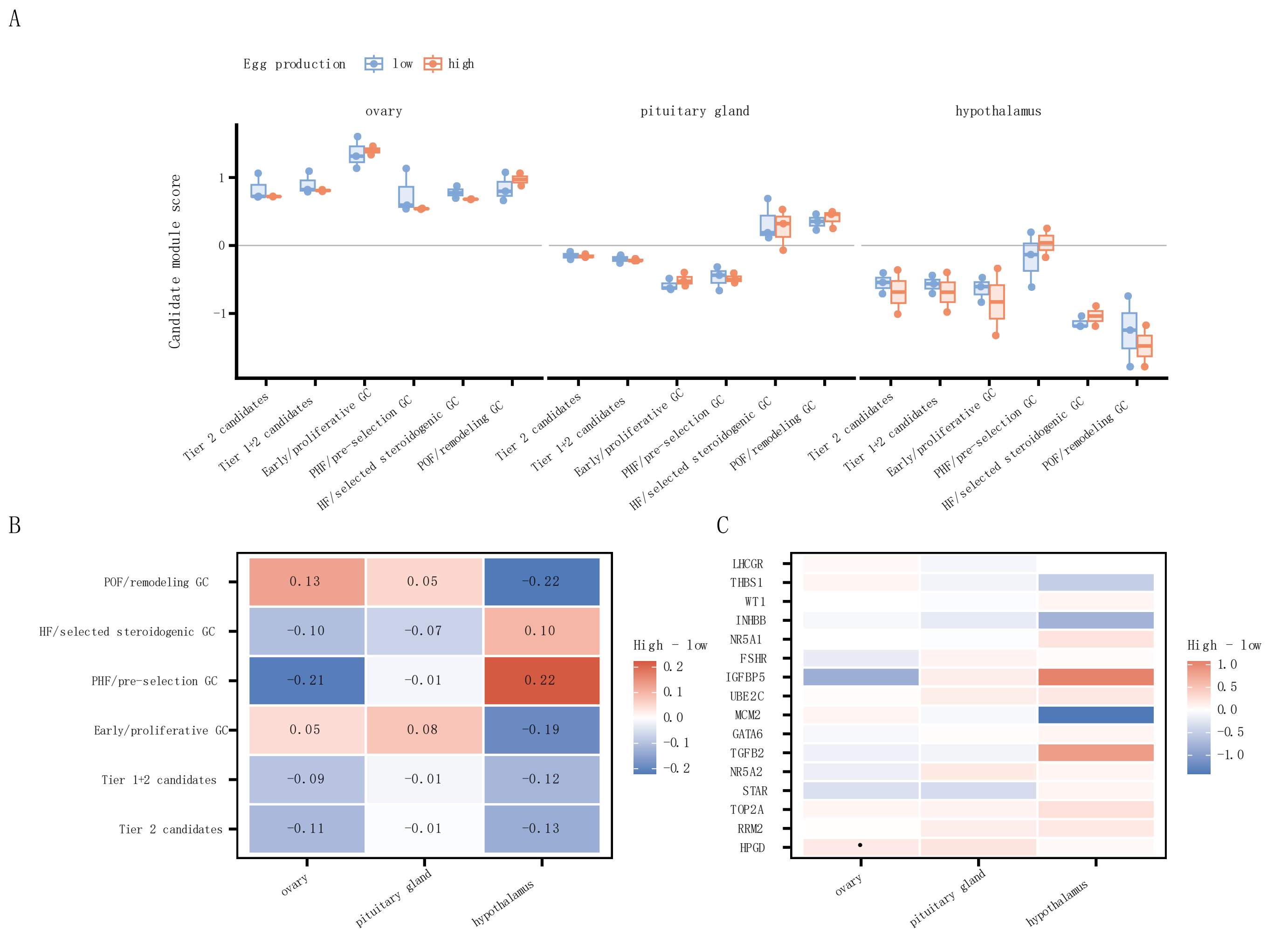

A
Egg production
low
high
ovary
pituitary gland
hypothalamus
Candidate module score
1
0
−1
Tier 2 candidates
Tier 1+2 candidates
Early/proliferative GC
PHF/pre-selection GC
HF/selected steroidogenic GC
POF/remodeling GC
B
POF/remodeling GC
HF/selected steroidogenic GC
PHF/pre-selection GC
Early/proliferative GC
Tier 1+2 candidates
Tier 2 candidates
0.13
0.05
−0.22
−0.10
−0.07
0.10
−0.21
−0.01
0.22
0.05
0.08
−0.19
−0.09
−0.01
−0.12
−0.11
−0.01
−0.13
ovary
pituitary gland
hypothalamus
High − low
0.2
0.1
0.0
−0.1
−0.2
C
LHCGR
THBS1
WT1
INHBB
NR5A1
FSHR
IGFBP5
UBE2C
MCM2
GATA6
TGFB2
NR5A2
STAR
TOP2A
RRM2
HPGD
ovary
pituitary gland
hypothalamus
High − low
1.0
0.5
0.0
−0.5
−1.0
